# Clinical significance of age at diagnosis among patients with thymic epithelial tumors: a population-based study

**DOI:** 10.18632/aging.102897

**Published:** 2020-03-30

**Authors:** Mina Liu, Changlu Wang, Lanting Gao, Changxing Lv, Xiaolong Fu

**Affiliations:** 1 Department of Radiation Oncology, Shanghai Chest Hospital, Shanghai Jiao Tong University, Shanghai 200030, China

**Keywords:** thymic epithelial tumors, age, pediatric tumors, survivals

## Abstract

Background: To investigate the clinicopathologic characteristics and survival outcomes of patients with thymic epithelial tumors (TET) according to age at diagnosis.

Results: A total of 4431 patients were analyzed. Gender, race, tumor histology and surgery were similar between different age groups. The 0-18 group was associated with a higher risk of distant metastasis. Compared to patients aged above 80, the hazard ratios (HR) for patients aged 0-18, 19-30, 31-40, 41-50, 51-60, 61-70, 71-80 were 1.079, 0.739, 0.614, 0.621, 0.633, 0.673, 0.861, respectively. From the subgroup analysis for the adult patients who were above 19 years old, we found that the 19-70 group had significant better cancer specific survival (CSS) and overall survival (OS) than the above 70 group.

Conclusions: Age is a strong independent prognostic factor for survival in TET. Pediatric TET has a higher risk of distant metastasis and an inferior CSS. For the adults who were above 19, patients older than 70-year-old were associated with a shorter CSS.

Methods: Information of 4431 TET patients was retrieved from the Surveillance, Epidemiology, and End Results (SEER) database. Demographic features, clinicopathologic characteristics and survival outcomes were compared between patients diagnosed at different age groups (0-18, 19-30, 31-40, 41-50, 51-60, 61-70, 71-80, above 80).

## INTRODUCTION

Thymic epithelial tumors (TET) originate in the thymus, including thymomas and thymic carcinomas. They constitute 0.2-1.5% of all malignancies [1]. The 5-year survival rate is approximately 90% [2] for thymomas and 55% for thymic carcinoma [3]. These tumors typically occur in adults with a median age of 50 [4], and rarely in children or adolescents. A cohort study from the European Society of Thoracic Surgeons database analyzed 2151 patients with thymic tumors from 35 institutions and showed that predictors of shorter overall survival (OS) included increased age [5]. However, the effect of age on TET is still controversial. The main reason may be the fact that the ranges of age of patients included in different published reports were various. Whether different age groups have homogeneous clinical features and survival outcomes remains unexplored. This study aimed to compare clinicopathologic characteristics and survival outcomes in different age groups using the Surveillance, Epidemiology, and End Results (SEER) database.

## RESULTS

A total of 4431 TET patients were identified from 1973 to 2014. The median age at diagnosis was 60. Twenty-eight (0.6%) patients were aged 1-18, 178 (4.0%) were aged 19-30, 381 (8.6%) were aged 31-40, 742 (16.7%) were aged 41-50, 971 (22.0%) were aged 51-60, 1108 (25.0%) were aged 61-70, 736 (16.6%) were aged 71-80 and 287 (6.5%) were aged above 80. The demographic and clinicopathological variables of the 4431 patients were listed in Table 1. Gender, race, histology and surgery were comparable among the age groups. Stage distribution was different. The proportions of Stage IVb diseases in the 0-18, 19-30, 31-40, 41-50, 51-60, 61-70, 71-80, and above 80 groups were 46.4%, 34.3%, 29.4%, 27.6%, 29.9%, 26.7%, 26.2% and 27.2%, respectively.

**Table 1:** The demographic and clinicopathological variables of the 4431 patients.

**Variable**	**No.**	**0-18 years (%)**	**19-30 years (%)**	**31-40 years (%)**	**41-50 years (%)**	**51-60 years (%)**	**61-70 years (%)**	**71-80 years (%)**	**81-90 years (%)**	**P value**
**Gender**										0.586
**Male**	2406	15 (53.6%)	93 (52.2%)	218 (57.2%)	405 (54.6%)	545 (56.1%)	581 (52.4%)	388 (52.7%)	161 (56.1%)	
**Female**	2025	13 (46.4%)	85 (47.8%)	163 (42.8%)	337 (45.4%)	426 (43.9%)	527 (47.6%)	348 (47.3%)	126 (43.9%)	
**Ethnicity**										0.332
**White**	3085	19 (67.9)	123 (69.1%)	266 (69.8%)	507 (68.3%)	656 (67.6%)	786 (80.0%)	525 (71.3%)	203 (70.7%)	
**Black**	619	7 (25%)	19 (10.7%)	61 (16%)	114 (15.4%)	147 (15.1%)	139 (12.5%)	93 (12.7%)	39 (13.6%)	
**Others**	703	2 (7.1%)	33 (18.5%)	53 (13.9%)	118 (15.9%)	161 (16.6%)	180 (16.2%)	112 (15.2 %)	44 (15.3%)	
**Unknown**	24	0	3 (1.7%)	1 (0.3%)	3 (0.4%)	7 (0.7%)	3 (0.3%)	6 (0.8%)	1 (0.4%)	
**Histology**										0.771
**Thymoma**	3512	20 (71.4%)	138 (77.5%)	297 (78.0%)	597 (80.5%)	777 (80.0%)	878 (79.2%)	573 (77.9%)	232 (80.8%)	
**Type A**	211	2	11	19	34	48	38	46	13	
**Type AB**	381	3	13	22	50	106	93	62	32	
**Type B1**	322	2	9	28	50	76	79	57	21	
**Type B2**	321	2	12	37	48	72	75	55	20	
**Type B3**	452	3	21	35	89	93	118	73	20	
**NOS**	1825	8	72	156	326	382	475	280	126	
**Thymic carcinoma**	919	8 (28.6%)	40 (22.5%)	84 (22.0%)	145. (19.5%)	194 (20.0%)	230 (20.8%)	163 (22.1%)	55 (19.2%)	
**Masaoka-Koga** **Stage**										0.000
**I-Iva**	2242	10 (35.7%)	74 (41.6%)	174 (45.7%)	351 (47.3%)	477 (49.1%)	627 (56.6%)	384 (52.2%)	145 (50.5%)	
**IVb**	1248	13 (46.4%)	61 (34.3%)	112 (29.4%)	205 (27.6%)	290 (29.9%)	296 (26.7%)	193 (26.2%)	78 (27.2%)	
**Unknown**	941	5 (17.9%)	43 (24.1%)	95 (24.9%)	186 (25.1%)	204 (21.0%)	185 (16.7%)	159 (21.6%)	64 (22.3%)	
**Surgery**										0.860
**Yes**	2973	22 (78.6%)	115 (64.6%)	266 (69.8%)	494 (66.6%)	655 (67.5%)	732 (66.1%)	504 (68.5%)	185 (64.5%)	
**No**	1384	6 (21.4%)	60 (33.7%)	108 (28.4%)	233 (31.4%)	300 (30.9%)	355 (32.0%)	223 (30.3 %)	99 (34.5%)	
**unknown**	74	0	3 (1.7%)	7 (1.8%)	15 (2.0%)	16 (1.6%)	21 (1.9%)	9 (1.2%)	3 (1.0%)	

### Survival analysis

A total of 2198 patients died, and among them, 1098 patients died of TET. The 3-, 5- and 10- year CSS rates were 85.1%, 81.4% and 77.5%, respectively. The 3-, 5- and 10- year OS rates were 74.9%, 67.5% and 57.4%, respectively. The survival conditions were listed in Table 2.

**Table 2:** The OS and the CSS of patients according to different histological types.

**OS**	**0-18**	**19-30**	**31-40**	**41-50**	**51-60**	**61-70**	**71-80**	**81-90**	**Median OS**
**Thymoma A**	47	not reached	144	191	80	98	47	46	82
**Thymoma AB**	9	98	238	241	97	81	57	51	88
**Thymoma B1**	147	131	not reached	108	114	93	53	25	103
**Thymoma B2**	27	114	140	126	160	78	76	57	111
**Thymoma B3**	78	100	124	155	145	121	64	45	112
**Thymic Carcinoma**	7	61	154	146	112	99	53	49	91
**CSS**	**0-18**	**19-30**	**31-40**	**41-50**	**51-60**	**61-70**	**71-80**	**81-90**	**Median CSS**
**Thymoma A**	47	not reached	not reached	not reached	not reached	161	not reached	not reached	not reached
**Thymoma AB**	9	98	238	241	280	not reached	not reached	not reached	280
**Thymoma B1**	147	not reached	not reached	188	174	not reached	not reached	not reached	188
**Thymoma B2**	27	240	not reached	276	not reached	not reached	not reached	not reached	276
**Thymoma B3**	78	140	not reached	263	272	not reached	not reached	103	276
**Thymic Carcinoma**	7	107	214	226	304	not reached	137	not reached	226

Multivariate survival analysis adjusted for gender, race, stage and histology showed that age was an independent prognostic factor for both OS (*p*<0.001) and cancer specific survival (CSS) (*p*=0.001). The results of the univariate and multivariate analysis of CSS for TET were listed in Table 3. Compared to patients aged above 80, the hazard ratios for patients aged 0-18, 19-30, 31-40, 41-50, 51-60, 61-70, 71-80 were 1.079 (95% CI: 0.621-1.875), 0.739 (95% CI: 0.521-1.047), 0.614 (95% CI: 0.452-0.835), 0.621 (95% CI: 0.469-0.822), 0.633 (95% CI: 0.481-0.832), 0.673 (95% CI: 0.512-0.885), 0.861 (95% CI: 0.648-1.143), respectively. Subgroup analysis showed that age was an independent prognostic factor for CSS (*p*=0.003) in patients with thymoma, but not a significantly independent predictor in patients with thymic carcinoma (*p*=0.079).

**Table 3:** Univariate and multivariate analysis of prognostic factors in TET.

**Variable**	**Univariate**		**Multivariate**	
	**HR (95% CI)**	**P value**	**HR (95% CI)**	**P value**
**Age group**		0.001		0.001
**0-18**	1.102 (95% CI: 0.633-1.916)	0.732	1.079 (95% CI: 0.621-1.875)	0.787
**19-30**	0.759 (95% CI: 0.535-1.076)	0.121	0.739 (95% CI: 0.521-1.047)	0.088
**31-40**	0.617 (95% CI: 0.454-0.838)	0.002	0.614 (95% CI: 0.452-0.835)	0.002
**41-50**	0.629 (95% CI: 0.475-0.833)	0.001	0.621 (95% CI: 0.469-0.822)	0.001
**51-60**	0.637 (95% CI: 0.484-0.838)	0.001	0.633 (95% CI: 0.481-0.832)	0.001
**61-70**	0.679 (95% CI: 0.516-0.893)	0.006	0.673 (95% CI: 0.512-0.885)	0.005
**71-80**	0.867 (95% CI: 0.653-1.152)	0.326	0.861 (95% CI: 0.648-1.143)	0.301
**Above 80**	1		1	
**Gender**		0. .972		/
**Male**	1		/	/
**Female**	0.998 (95% CI:0.885-1.125)		/	/
**Ethnicity**		0.207	/	/
**White**	1		/	/
**Others**	0.983 (95% CI: 0.827-1.168)	0.844	/	/
**Black**	0.833 (95% CI: 0.700-0.990)	0.039	/	/
**Unknown**	0.773 (95% CI: 0.320-1.866)	0.567	/	/
**Histology**		0.414		
**Thymoma**	0.941 (95% CI: 0.814-1.088)		/	/
**Thymic carcinoma**	1		/	/
**Masaoka-Koga** **Stage**		0.000		0.000
**I-Iva**	1			
**IVb**	5.240 (95% CI: 4.495-6.108)	0.000	5.235 (95% CI: 4.491-6.103)	0.000
**Unknown**	2.759 (95% CI: 2.334-3.261)	0.000	2.756 (95% CI: 2.332-3.257)	0.000
**Surgery**		0.241		
**Yes**	0.952 (95% CI: 0.838-1.082)	0.455	/	/
**No**	1		/	/
**unknown**	0.644 (95% CI: 0.377-1.099)	0.107	/	/

**Figure 1:**
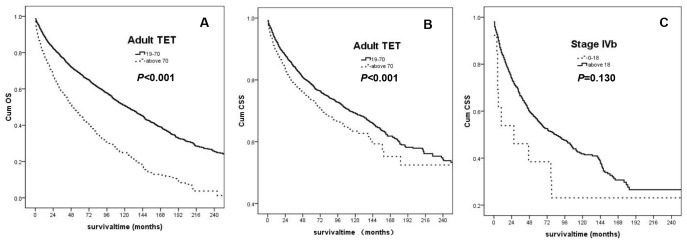
(**A**) OS for the adult TET Patients (the 19-70 age group: solid line; the above 70 group: dashed line); (**B**) CSS for the adult TET patients (the 19-70 age group: solid line; the above 70 group: dashed line); (**C**) CSS for patients with the stage IV b (the 0-18 age group: dashed line; the above 18 group: solid line).

According to the results in Cox proportional hazards regression analysis, we divided the adult patients into two groups, the 19-70 group and the above 70 group. From the subgroup analysis, we found that the younger group had significant better CSS and OS than the older group (CSS: p<0.001; OS: p<0.001, Figure 1A and 1B).

Subgroup analysis also showed that in patients with IVb TET, the 0-18 group had a tendency towards inferior CSS compared with the adult patients (p=0.130). The Kaplan-Meier curve was demonstrated in Figure 1C. There were 265 patients diagnosed with stage IVb thymoma, including 5 aged under 18 years old and 260 aged above 18. The median CSS was 6 months for the younger patients and 90 months for the older ones. There were 983 patients diagnosed with stage IVb thymic carcinoma, including 8 aged under 18-year-old and 975 above 18. The median CSS was 47 months for the younger group and 76 months for the older group, respectively. Totally, the median CSS was 27 months for the 0-18 group, and 84 months for the adults.

### DISCUSSION

The effect of age on survival in TET remains controversial. Ruffini E et al. demonstrated that increased age was one of the predictors of shorter OS in thymic carcinoma [5]. A retrospective analysis on 797 thymoma patients showed that age, incomplete resection, and advanced stages were negative prognostic variables [6]. However, in contrast, Rea F et al. displayed that only WHO histology and Masaoka stage were identified as independent prognostic factors in the multivariate analysis including factors of gender, age, Myasthenia Gravis (MG) and other parathymic syndromes, adjuvant radiotherapy, extent of resection, Masaoka stage, WHO histology and recurrence [7]. Gripp S et al. found age had no effect on survival outcomes for patients with thymoma [8]. A single-center analysis also showed that R0 resection was the only independent prognosticator of OS [9]. The retrospective design and the limited sample size may be the possible reasons. The primary factor contributed to the discrepancy was that the ranges of age in different published reports were various. Therefore, in our study, we included patients from 0-year-old to 94-year-old and found that age was one of the strong prognostic factors for both OS (*p*=0.001) and CSS (*p*=0.001) for patients with TET.

Although OS is the most often reported measure of survival probability, CSS may be more meaningful for TET because it excludes non-TET causes of deaths and provides more disease-specific prognosis [10]. In the multivariate analysis to assess the prognostic factors for CSS, patients in the 31-40, the 41-50, the 51-60 and the 61-70 age groups had significantly better CSS compared with the above 80 age group, with the HRs of 0.614 (95% CI: 0.452-0.835), 0.621 (95% CI: 0.469-0.822), 0.633 (95% CI: 0.481-0.832), and 0.673 (95% CI: 0.512-0.885). No significant difference existed when comparing the above 80 group with either the 0-18 or the 19-30 group or the 71-80 age group. For the adults, we found that although patients beyond 70-year old had similar stage distributions as the younger adults (19-70 years old; *p*=0.677), they have significantly inferior OS (*p*<0.001) and CSS (*p*<0.001). Older patients are associated with more comorbidities and poorer performance status. They may not be able to tolerate radical surgery or radiotherapy or intense chemotherapy and have a higher risk of treatment-related deaths.

Pediatric TET was associated with an increased risk of distant metastasis. Nearly half of the pediatric patients of TET in our study were diagnosed of metastatic diseases. Even in the same stage, the pediatric patients had a tendency towards inferior survival compared with the adult counterparts. For pediatric patients with stage IVb diseases, the CSS was only 27 months. However, the CSS was 84 months for the adults (*p*=0.130). Previous studies reported that pediatric thymomas had a slight male predominance [11]. However, our study showed no significant difference in gender existed among different age groups. Also, the distribution of races and histology were similar. These findings suggest pediatric TET exhibits distinct biological behavior. It is indicated that TET has a more aggressive biologic behavior in young patients than in older-aged patients. Molecular mechanisms underlying childhood and adolescent TET have been explored [12–14]. Various genetic background, such as Human Leucocyte Antigens (HLA) genotyping, may be associated with the early onset and distinctive prognosis of the subgroup. The physiological changes accompanied with aging, especially the immune system, may be another reason. The immune functions associated with thymus are different between the adults and the younger individuals. Although there was still insufficient explanation regarding this issue, we hope the results of our study will encourage more research in this field.

This study has several limitations. First, it is a retrospective study which had an unavoidable selection bias. Furthermore, patients’ performance status and comorbidities, pathologic resection margins, the use of chemotherapy or radiotherapy and surgery skills are not reported in the SEER database. In addition, the exact stage information was unknown in SEER. The Masaoka stage in the study was inferred from several existing variables. Therefore, further studies are required to validate our findings.

### MATERIALS AND METHODS

This retrospective study was based on publicly available SEER database. Data were retrieved through online access using the SEER*Stat software Version 8.2.1. The Institutional Review Board of our Hospital approved this study.

### Data collection

We used SEER database 1973-2014 that was submitted in November 2016. Cases with the primary site of the thymus were obtained using the variable of "primary site labeled" and the histology were determined by the International Classification of Diseases Codes (Thymoma: 8580-8585; Thymic carcinoma: 8011, 8020, 8050, 8052, 8070-8072, 8074, 8082, 8083, 8094, 8123, 8140, 8260, 8310, 8430, 8480, 8560, and 8586). Individual data retrieved for each case included age at diagnosis, gender, race, year of diagnosis, tumor histology, SEER staging information, treatments (surgery/lymph nodes removed/ lymph nodes positive), cause-specific death classification, vital status and survival months.

Because patients' stage information was unknown in SEER, the stage information was inferred from several existing variables, such as primary tumor extension, lymph node status, and SEER historic stage. Positive lymph node disease or the "distant" status of the SEER stage was considered as stage IVb [15]. “Local” or “Regional” status of the SEER stage with no lymph node metastasis was considered as stage I-Iva. “Regional” status with unknown lymph node condition or “unknown” status was considered as stage unknown.

### Statistical analysis

In this study, patients were stratified into eight subgroups (0-18, 19-30, 31-40, 41-50, 51-60, 61-70, 71-80, above 80) according to age at diagnosis. The Chi-square test and the Fisher’s exact probability test were used to compare demographic and clinicopathological variables between age groups. Kaplan-Meier curves were estimated and compared using the log-rank test. Multivariate survival analysis was performed using the Cox proportional hazards regression to identify independent prognostic factors. Hazard ratio (HR) and its 95% confidence interval (CI) were calculated. Statistical analysis was conducted in SPSS 16.0 (SPSS Inc, Chicago, IL). All tests were two tailed, and *p*<0.05 was considered as statistically significant.
